# Plasma Interleukin-33 Cannot Predict Hip Osteonecrosis in Patients With Sickle Cell Disease: A Case-Control Study

**DOI:** 10.7759/cureus.23556

**Published:** 2022-03-28

**Authors:** Alok C Agrawal, Eli Mohapatra, Rachita Nanda, Narendra Kuber Bodhey, Harshal Sakale, Ankit Kumar Garg

**Affiliations:** 1 Orthopaedics, All India Institute of Medical Sciences, Raipur, Raipur, IND; 2 Biochemistry, All India Institute of Medical Sciences, Raipur, Raipur, IND; 3 Radiodiagnosis, All India Institute of Medical Sciences, Raipur, Raipur, IND

**Keywords:** plasma interleukin-33 level, hip, avn, sickle cell disease, osteonecrosis of the femoral head

## Abstract

Background

Plasma interleukin-33 (IL-33), a cytokine associated with inflammatory and autoimmune disease, has been described to be significantly raised in osteonecrosis of the femoral head (ONFH) and hence was recommended for use as a marker for ONFH. The concentration of plasma interleukin-33 level has not been estimated in any studies conducted in patients with sickle cell disease (SCD); hence, we investigated the levels of plasma interleukin-33 in patients with sickle cell disease with or without ONFH to assess whether it can be used as a marker for the early detection of ONFH in this disease also.

Methods

Forty-four consecutive patients with sickle cell disease with osteonecrosis of the femoral head and matched controls without ONFH were evaluated for plasma interleukin-33 levels by enzyme-linked immunosorbent assay (ELISA). All patients were confirmed for sickle cell disease using high-performance liquid chromatography (HPLC). ONFH was diagnosed in patients with sickle cell disease using clinical-radiological findings. Univariate and multivariate analyses were performed using the IL-33 level as the dependent variable.

Results

Plasma IL-33 levels were comparable in 44 patients with sickle cell disease with osteonecrosis of the femoral head as compared with 24 patients with sickle cell disease without ONFH (2.05 ± 4.57 pg/mL versus 1.50 ± 2.89 pg/mL, p-value = 0.590). There was no significant difference in IL-33 levels in different stages of avascular necrosis (AVN).

Conclusions

Plasma interleukin-33 levels cannot act as a marker of ONFH as were being considered in idiopathic ONFH or ONFH caused by other causes such as trauma and chronic steroid or alcohol usage.

## Introduction

Osteonecrosis of the femoral head (ONFH) is a disabling condition of young individuals. Its etiology and pathogenesis are not completely known. In its natural course, 70%-80% of patients develop secondary hip joint osteoarthritis [1]. Trauma, including a fractured neck of the femur, is considered a major cause of ONFH. Nontraumatic ONFH can be caused by prolonged use of corticosteroids, chronic alcohol consumption, infections, hyperbaric damage to the vascularity of the femoral head, glycogen and lipid storage disorders, marrow infiltrating diseases, coagulation defects, very low or high temperatures, and some autoimmune diseases [2]. Sickle cell anemia or sickle cell disease (SCD) is also common in some parts of the world, including India, and is a common cause of ONFH. The incidence of osteonecrosis of the femoral head has been described to be 22% in patients with sickle cell disease [3] and up to 50% in some other series [4]. In this disease, valine substitutes instead of glutamic acid in the beta chain of red blood corpuscles (RBCs) at the genetic level. This predisposes RBCs to become sickle-shaped when oxygen saturation becomes less in the circulating blood, and this, in turn, is known to cause an increased incidence of avascular necrosis (AVN) of the femoral head [4]. The incidence of ONFH has been described as more in the homozygous SS sickle cell disease than in the sickle cell trait, in which it is described as rare [5].

The prevalence of sickle cell disease is estimated at about 2.1% in Chhattisgarh, and that of the sickle cell trait is about 10%. The state of Chhattisgarh is endemic to sickle cell disease [6]. It can simultaneously affect multiple joints; the femoral head is more vulnerable as it lacks collateral blood flow. The most common cause of ONFH in Central India is sickle cell disease (SCD). The abnormal sickle-shaped adherent red blood cells cause ischemia of the femoral head articular cartilage, leading to bone infarction at the epiphysis, and early-onset degenerative arthritis [5,7-10].

Interleukin-33 (IL-33) is a cytokine associated with inflammatory and autoimmune diseases. It is a member of the IL-1 family of cytokines discovered in 2007 [11]. IL-33, a pro-inflammatory cytokine, is released from osteonecrotic bones. It is mainly released from osteoblasts, adipocytes, and osteocytes by cells undergoing necrosis rather than by active secretion [12-15]. IL-33 is believed to act as a primary inflammatory cytokine in septic shock, proliferative diseases, asthma, cardiovascular diseases, collagen vascular diseases, and pleural malignancies [16]. During ONFH, IL- 33 also plays a role by directly and indirectly impacting bone remodeling after it is released from osteonecrosis of bones [17]. The origin, mode of action, and function of interleukin-33 are still under research, as well as whether IL-33 increases with the repair of necrotic bone or whether IL-33 acts as a positive or a negative effect after ONFH.

Plasma interleukin-33 levels have been described to be significantly raised in osteonecrosis of the femoral head, which may be steroid-induced, alcohol-induced, secondary to an infection, coagulation defects, storage disorders, marrow infiltrating diseases, autoimmune diseases, immoderately low or high temperatures, hyperbaric events, or idiopathic disease. IL-33 is raised in all of these conditions with ONFH and was recommended for use as a marker for osteonecrosis of the femoral head [18,19].

The concentration of plasma interleukin-33 levels has not been estimated in patients with sickle cell disease; hence, we investigated the levels of plasma interleukin-33 in adult patients with sickle cell disease with or without ONFH to assess whether its levels can be used as a marker for the early detection of the disease.

## Materials and methods

Study design

The present study is a case-control study.

Study population

After obtaining institutional review board approval (OW-55/AIIMS-RPR/RC/2017) on October 28, 2017, from All India Institute of Medical Sciences (AIIMS), Raipur, and written informed consent, we included patients presenting to tertiary referral center during the period from September 2017 to December 2020. All consecutive patients with age between 18 and 70 years with known sickle cell disease with a history of hip pain and limping were included in the study. Patients with a history of significant trauma, inflammatory disease, active infection on the affected hip, immunodeficiency, HIV infection, history of long-term steroid use for any illness, or previous surgery on the hip were excluded from the study. The patients were diagnosed with osteonecrosis of the femoral head (ONFH) based on clinical history, physical examination, and radiological evaluation (X-ray and MRI) by the orthopedic surgeons. We confirmed the diagnosis of all patients with sickle cell disease with electrophoresis and HPLC. A total of 44 patients were diagnosed to have osteonecrosis of the femoral head (ONFH).

Control group

Twenty-four age- and sex-matched healthy subjects with known sickle cell disease were simultaneously recruited as controls. All participants in the control group were examined clinically and radiologically to rule out any hip disease in order to prevent selection bias.

Staging

All patients with osteonecrosis of the femoral head (ONFH) were evaluated according to the China-Japan Friendship Hospital (CJFH) classification [20]. The CJFH classification is based on the area of involvement of necrosis in the three pillars (medial (M), central (C), and lateral (L)) seen in the mid-coronal image of MRI/CT scan and the intact degree of the lateral pillar (L1, L2, and L3).

Methodology for IL-33 measurements

A blood sample (3 mL) was collected in sterile anticoagulation (heparin) tubes from the cases and controls. Then, the samples were sent to the laboratory for the estimation of IL-33. It was centrifuged at 3,000 rpm for 15 minutes to separate plasma. The plasma was immediately frozen and stored at -80°C for analysis at a later date. Plasma interleukin-33 was to be detected by enzyme-linked immunosorbent assay (ELISA). All samples and standards were run in duplicate. The average absorbance values for each set of duplicate standards and samples were calculated. A standard curve was plotted using the mean absorbance for each standard concentration and concentrations of IL-33 for each patient.

Statistical analysis

Data were analyzed using SPSS version 24.0 (IBM Corporation, Armonk, NY, USA). Quantitative variables were reported as mean ± standard deviation (SD). Nonpaired t-tests were used to compare the IL-33 levels in both groups. For statistically significant differences, groups were to be compared using the least significant difference (LSD) test. All tests were to be two-tailed at the 5% level of significance.

Funding

The study was funded by an intramural grant given by the employer institute for the purpose of research.

## Results

Demographic data

In our study, 44 patients with sickle cell disease had osteonecrosis of the femoral head (M/F = 29/15) as compared with 24 patients in the control group (M/F = 9/13). The mean age was comparable between the groups, 26.2 ± 8.23 years in the case group and 28.08 ± 10.89 years in the control group (Table 1).

**Table 1:** Demographic data of the two groups

Demographics of the patients	Case (ONFH) (number/mean (SD))	Control (number/mean (SD))	P-value
Total number of patients	44	24	
Age (years)	26.2 ± 8.23	28.08 ± 10.89	0.469
Sex			
Male	29	9	0.037
Female	15	13	
CJFH classification			
Type M	1		
Type C	7		
Type L	36		
Type L1	4		
Type L2	15		
Type L3	17		

Among the 44 patients with ONFH in the case group, the CJFH types were as follows: type M in one hip, type C in seven hips, and type L in 36 hips (L1 = 4, L2 = 15, and L3 = 17).

Plasma IL-33 levels

Plasma interleukin-33 levels, which were examined using ELISA, were demonstrated to be 2.05 ± 4.57 pg/mL and 1.50 ± 2.89 pg/mL in patients with sickle cell disease with ONFH and without ONFH, respectively (Table 2).

**Table 2:** Comparison between the two groups

Variables	Case	Control	P-value
IL-33 level (pg/mL)	2.05 ± 4.57	1.50 ± 2.89	0.590
Hb A	15.04 ± 21.76	49.83 ± 17.87	<0.001
Hb A_2_	2.48 ± 0.93	3.09 ± 0.61	0.006
Hb F	17.96 ± 6.87	6.28 ± 9.03	0.001
Hb S	63.72 ± 15.33	37.52 ± 12.52	<0.001
Hb	10.7 ± 1.8	11.4 ± 2.14	0.99

There was no significant difference in plasma interleukin levels among patients with sickle cell disease with ONFH and without ONFH (p-value = 0.590).

Many literature reviews have suggested that ONFH is associated with homozygous patients with sickle cell disease, but in our study, we found six heterozygous patients with sickle cell trait that is associated with ONFH [2,3]. A significant difference was found on HPLC, whereby the Hb S and HB F levels were significantly higher in ONFH. There was no significant difference in Hb levels in patients with sickle cell disease with or without ONFH. The levels were normal (Table 2).

**Figure 1:**
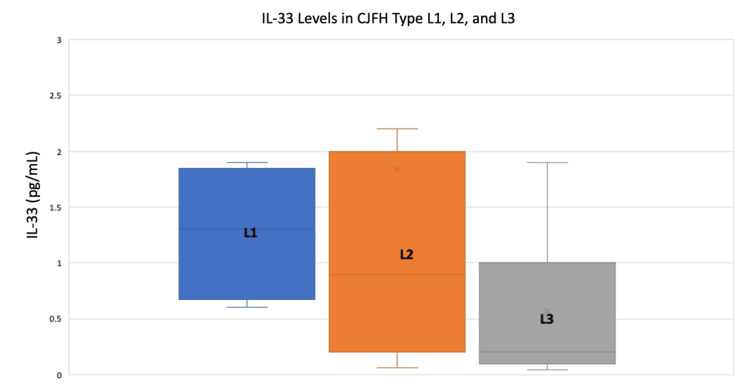
Plasma IL-33 levels in ONFH patients with CJFH types L1, L2, and L3 (1.27 ± 0.62 pg/mL, 1.24 ± 1.40 pg/mL, and 1.08 ± 2.46 pg/mL, respectively)

The levels of plasma IL-33 were 1.27 ± 0.62 pg/mL, 1.24 ± 1.40 pg/mL, and 1.08 ± 2.46 pg/mL in patients with ONFH CJFH types L1, L2, and L3, respectively (Figure 1). There was no significant difference between the groups (p-value = 0.968).

## Discussion

Several studies decide whether interleukin-33 can be taken up as a marker of avascular necrosis of the femoral head [18,19]. Plasma interleukin-33 levels correlated well with ONFH, but these studies did not mention sickle cell disease as a cause of osteonecrosis. So, it was not clear whether plasma interleukin-33 levels were relevant in patients with sickle cell disease or not. To the best of our knowledge, no study has been done until now on patients with sickle cell disease, and our study was planned to investigate the plasma levels of IL-33 in patients with sickle cell disease and in patients with sickle cell disease with ONFH.

Earlier studies have described the generally considered direct effects of IL-33 on the osteoclasts and its rising level in the circulation after its release from the necrotic cells, but the effect of IL-33 in patients with sickle cell disease with ONFH has not been described. Our study found that plasma interleukin-33 levels were comparable in patients with sickle cell disease with or without ONFH. Also, interleukin-33 levels were comparable in different stages of ONFH as classified by the CJFH classification. As the levels of plasma interleukin-33 in patients with sickle cell disease are always low, the level need not be correlated with ONFH grade. This comes as a novel finding since many studies have described an increased level of IL-33 in patients with ONFH.

A study conducted by Zheng et al. on 125 patients with ONFH and 126 controls irrespective of the cause of ONFH found that plasma IL-33 levels were higher in patients with ONFH and increased with the increase in the stage of the disease [18]. Another study done by Ma et al. on 40 patients with ONFH, irrespective of the cause, also corroborated the above study and found that plasma IL-33 levels were higher in patients with ONFH [19].

The present study has some limitations. Firstly, the sample size in the control group is relatively small. Secondly, we have not analyzed the relationship between necrotic area and the levels of plasma IL-33 in ONFH. Thirdly, the study should be planned on a larger population as the incidence of sickle cell disease is 2.1 % for homozygous cases and 10 % for sickle cell traits.

## Conclusions

Plasma IL-33 levels do not correlate with osteonecrosis of the femoral head in patients with sickle cell disease. However, we recommend further studies examining local cytokine signaling for specific biological phenomena such as osteonecrosis of the femoral head.

## References

[1] Tripathy SK, Goyal T, Sen RK (2015). Management of femoral head osteonecrosis: current concepts. Indian J Orthop.

[2] Assouline-Dayan Y, Chang C, Greenspan A, Shoenfeld Y, Gershwin ME (2002). Pathogenesis and natural history of osteonecrosis. Semin Arthritis Rheum.

[3] Adesina O, Brunson A, Keegan TH, Wun T (2017). Osteonecrosis of the femoral head in sickle cell disease: prevalence, comorbidities, and surgical outcomes in California. Blood Adv.

[4] Fitzsimmons R, Amin N, Uversky VN (2016). Understanding the roles of intrinsic disorder in subunits of hemoglobin and the disease process of sickle cell anemia. Intrinsically Disord Proteins.

[5] Acurio MT, Friedman RJ (1992). Hip arthroplasty in patients with sickle-cell haemoglobinopathy. J Bone Joint Surg Br.

[6] Thakur S, Sharma R, Sharada RN (2014). Incidence of thalassemia and sickle cell disease in Chhattisgarh, Central India: using Hardy-Weinberg equations. J Mol Genet Med.

[7] da Silva Junior GB, Daher Ede F, da Rocha FA (2012). Osteoarticular involvement in sickle cell disease. Rev Bras Hematol Hemoter.

[8] Poignard A, Flouzat-Lachaniette CH, Amzallag J, Galacteros F, Hernigou P (2012). The natural progression of symptomatic humeral head osteonecrosis in adults with sickle cell disease. J Bone Joint Surg Am.

[9] Flouzat-Lachaniete CH, Roussignol X, Poignard A, Mukasa MM, Manicom O, Hernigou P (2009). Multifocal joint osteonecrosis in sickle cell disease. Open Orthop J.

[10] Stoica Z, Dumitrescu D, Popescu M, Gheonea I, Gabor M, BO N (2009). Imaging of avascular necrosis of femoral head: familiar methods and newer trends. Curr Health Sci J.

[11] Carriere V, Roussel L, Ortega N (2007). IL-33, the IL-1-like cytokine ligand for ST2 receptor, is a chromatin-associated nuclear factor in vivo. Proc Natl Acad Sci U S A.

[12] Saidi S, Bouri F, Lencel P (2011). IL-33 is expressed in human osteoblasts, but has no direct effect on bone remodeling. Cytokine.

[13] Schulze J, Bickert T, Beil FT (2011). Interleukin-33 is expressed in differentiated osteoblasts and blocks osteoclast formation from bone marrow precursor cells. J Bone Miner Res.

[14] Saleh H, Eeles D, Hodge JM (2011). Interleukin-33, a target of parathyroid hormone and oncostatin m, increases osteoblastic matrix mineral deposition and inhibits osteoclast formation in vitro. Endocrinology.

[15] Nile CJ, Barksby E, Jitprasertwong P, Preshaw PM, Taylor JJ (2010). Expression and regulation of interleukin-33 in human monocytes. Immunology.

[16] Kakkar R, Lee RT (2008). The IL-33/ST2 pathway: therapeutic target and novel biomarker. Nat Rev Drug Discov.

[17] Saidi S, Magne D (2011). Interleukin-33: a novel player in osteonecrosis of the femoral head?. Joint Bone Spine.

[18] Zheng L, Wang W, Ni J (2014). Plasma interleukin 33 level in patients with osteonecrosis of femoral head: an alarmin for osteonecrosis of the femoral head?. J Investig Med.

[19] Ma J, Guo W, Li Z, Wang B, Li S, Wang P (2017). Hip osteonecrosis is associated with increased plasma IL-33 level. Mediators Inflamm.

[20] Zirong L, Liu Z, Sun W (2012). The classification of osteonecrosis of the femoral head based on the three pillars structure: China-Japan Friendship Hospital (CJFH) classification. Chinese J Ortho.

